# Patient Satisfaction and Comprehension of Physician and Pharmacist Prescription in Saudi Arabia: A Cross-Sectional Study

**DOI:** 10.7759/cureus.27324

**Published:** 2022-07-27

**Authors:** Ammar K Alharbi, Arwa A Alhutayrashi, Areen N Alosaimi, Sawsan M Althubyani, Mokhtar Shatla

**Affiliations:** 1 College of Medicine, Umm Al-Qura University, Makkah, SAU; 2 Medicine and Surgery, Umm Al-Qura University, Makkah, SAU; 3 Emergency Department, Prince Abdulaziz Bin Musaed Hospital, Arar, SAU; 4 Family Medicine, Umm Al-Qura University, Makkah, SAU

**Keywords:** patient satisfaction, comprehension, healthcare system, patient understanding, pharmacist, physician, medical prescription, complex medication regimen

## Abstract

Background and objectives

Patient satisfaction is a measure of patient quality of life, and the perspective and experience of patients would further improve different aspects of healthcare services of both the health plan and health care. This study aims to assess patient satisfaction with both physicians and pharmacists, as well as how well patients comprehended their prescriptions.

Methods

This was a cross-sectional, survey-based study conducted from January to April 2022 in Saudi Arabia. An online survey was spread through social media and was filled out over two months. The survey received 575 responses and it included participants' demographics and general satisfaction toward both physicians and pharmacists and healthcare services. Two modified tools, i.e., the Patient Satisfaction with Pharmacist Services Questionnaire (PSPSQ 2.0) and the Patient Satisfaction Questionnaire (PSQ-18), were used for this study.

Results

The participants were very satisfied (overall satisfaction: 82.5%) with their education on how to take medicine (87.1%), and the information was easy to understand (89.9%), they were able to ask for more information (67.7%), and the explanations were very clear (84.0%). The level of satisfaction toward healthcare services with both the physician and pharmacist was high (78% with a mean score of 50.7 ± 9.8). The self-perceived general satisfaction toward participant capacity and comprehension by bio-demographic data was higher in females than in males (3.34 ± 1.0 vs. 3.13 ± 1.14, respectively), with a statistically significant difference (p = 0.032). The satisfaction toward healthcare services with both physicians and pharmacists was higher in participants with low education levels compared with those with higher education levels (54.7 ± 8.3 vs. 50.3 ± 10.0; p = 0.049), respectively.

Conclusions

The current study demonstrated a high level of satisfaction among participants with healthcare services and both physicians and pharmacists in general. The majority of the participants were satisfied with the education they received, and they considered the information on how to take the drug to be simple to comprehend.

## Introduction

Satisfaction has become the ultimate tool for measuring patients' interaction with healthcare systems, and it is an important parameter in the evaluation of healthcare quality. It serves as a metric for assessing healthcare delivery and the healthcare system in general. Maintaining and sustaining an effective health system that is comprehensive and more patient-centered care that fulfills the patient's expectations, thereby reflecting their satisfaction and opinions about the healthcare system, is critical [1,2]. While there are no exact measures and consistent items that address and fulfill all patients' demands and thus satisfaction [3], several satisfaction studies have been conducted to include physicians' consultations, for example, length of consultation, professional care, and communications, in primary healthcare services in general, as well as other factors related to healthcare services such as distance health care and appointments [4]. However, only a few studies investigated patient satisfaction in healthcare delivery and the healthcare system in general. A variety of studies analyze the impact of physician practices, communication, and service access on patient satisfaction and how these relate to patients' continuity of medical care [5-10]. The aspect of care and patients' comprehension of their prescribed drugs have been incorporated in a few studies [11-14]. Patients' perceptions are taken into consideration when healthcare managers design strategies to improve the quality of care. Physicians and pharmacists must promote and ensure that drug label information is delivered and understood and that the required dose is administered correctly to achieve an excellent quality process [15].

Many patients who present with chronic diseases and comorbidities to primary health care are elderly and usually managed by family medicine specialists. Prescription of complex medication regimens is widespread in healthcare centers [16-18]. Thus, simplifying medication regimens and educating them in sufficient time and efficient communication help in increasing the satisfaction, perception, and continuity of medication and adherence [19-22]. A study conducted in Riyadh interviewed 511 adult participants and aimed to assess the understanding of prescription label instructions for five commonly prescribed medications and concluded that misunderstanding was common among these participants [11]. This might indicate that not all patients understand their prescription, and non-confirming adherence might be common [18]. In our study, we aimed to measure participants’ satisfaction with both physicians and pharmacists and assess their level of comprehension of medication prescription.

## Materials and methods

Study design

The study was a descriptive cross-sectional study that was conducted over a period of four months, from January to April 2022, using an electronic questionnaire spread on social media via data collectors from different regions in Saudi Arabia. Ethical approval was obtained from the Biomedical Research Ethics Committee of Umm Al-Qura University (HAPO-02-K-012-2022-02-987). Eligible participants were informed prior to filling out the questionnaire that they could decline to participate at any time and that they had the option to not complete the questionnaire. Researchers informed the eligible participants that they will remain anonymous to ensure confidentiality and that ethical approval was received. The researchers translated the questions from English to Arabic and back to English. The Arabic version of the survey was used to collect the data. The data were only available to the research team.

Inclusion and exclusion criteria

Patients who were at least 14 years of age, had at least one level of education, and had a visit to any healthcare facility were included, regardless of where or how the participants received their care. Participants had to complete all questions before submitting the survey, and they had to have at least one medication to complete the study and provide their level of satisfaction.

Data collection method and sampling technique

A sample size of 575 participants was collected using a non-probability sampling technique, and the survey was distributed to the public randomly from February 2022 to March 2022 on social media via eight data collectors situated in different regions in Saudi Arabia.

Questionnaire

The survey was made in January 2022 using a modified combination of two tools, i.e., the Patient Satisfaction with Pharmacist Services Questionnaire (PSPSQ 2.0) and the Patient Satisfaction Questionnaire (PSQ-18), and consisted of 23 items (Appendix). The questions were revised by the principal investigator and edited according to his suggestions. The questionnaire included 23 questions and was self-divided into the components listed below.

Patients’ Bio-Demographics

Participants' age, gender, education level, clinical condition, number of medications they were taking, and how long they had been taking these prescriptions were all taken into consideration. Participants had to pick from nine causes and conditions (hyperglycemia, hypertension, gastrointestinal, heart or lung, bones, urinary tract, eyes, skin, and tumors), and if their reason was not stated above, they had to provide their reason for the visit, and those who did were included.

*Self-Perceived Satisfaction Toward* *Patient Capacity and Comprehension*

This domain consisted of four items for which participants had to choose "yes" or "no" as an answer. The questions included if the participants had been educated on how to use the medication and whether the information given to them was easy to understand, were they capable of asking for more information about their prescription, and if all of their questions were answered clearly.

Patients’ Satisfaction With Physicians and Pharmacists in General and Healthcare Services

The study questions in this domain included 13 items related to participants' satisfaction toward both physicians and pharmacists, including communications, instruction, explanation, behavior and corporation, medication, and health services, availability and accuracy of the prescribed medications, and availability of health services. Participants had to choose between “disagree,” “neutral,” and “agree” in this domain.

Data analysis

After data extraction, data were revised, coded, and fed to statistical software IBM SPSS version 22 (IBM Corp., Armonk, NY). All statistical analyses were done using two-tailed tests. A p-value less than 0.05 was considered statistically significant. The overall score for the two satisfaction domains was calculated by adding discrete item scores for each domain (the general satisfaction toward the physician and pharmacist and self-perceived general satisfaction toward the patient capacity). All variables, including patients' socio-demographic data, prescriptions received, and drug intake duration, were subjected to descriptive analysis based on frequency and percent distribution. Additionally, participants' satisfaction scale items were displayed. The mean score for each satisfaction domain was determined, along with the standard deviation. A one-way ANOVA and an independent t-test were used to analyze the distribution of patient satisfaction levels based on their demographic data and prescriptions. The association between general satisfaction with physicians and pharmacists and self-perceived general satisfaction with the patient capacity was analyzed using correlation analysis.

## Results

A total of 575 patients fulfilling the inclusion criteria completed the study questionnaire. Patients' age ranged from 18 to 64 years with a mean age of 34.2 ± 11.6 years. Approximately 419 (72.9%) patients were females and 483 (84%) were university graduates. As for the number of drugs received, 482 (83.8%) patients received one to two drugs, 75 (13%) received three to five drugs, and 18 (3.1%) patients received more than five drugs. A total of 261 (45.4%) patients received the drugs for up to seven days, and 314 (54.6%) received the drugs for more than seven days (Table 1).

**Table 1:** Bio-demographic data of study patients

Bio-demographic data	No.	%
Age in years		
<25	184	32.0%
25-34	125	21.7%
35-44	109	19.0%
45-55	127	22.1%
>55	30	5.2%
Gender		
Male	156	27.1%
Female	419	72.9%
Educational level		
Below secondary	19	3.3%
Secondary	73	12.7%
University/above	483	84.0%
Number of received drugs		
1-2	482	83.8%
3-5	75	13.0%
>5	18	3.1%
Duration of using these drugs		
≤7 days	261	45.4%
>7 days	314	54.6%

Participants' conditions or reasons to visit the health care were as follows: diabetes (9%), hypertension (7.7%), gastrointestinal (17.2%), cardiopulmonary (11%), orthopedic/bone (12.9%), urological (2.6%), ophthalmological (9.6%), dermatological/skin (14%), and tumors (0.9%) (Table 2).

**Table 2:** Self-perceived general satisfaction toward patient capacity and comprehension among study patients

Self-perceived general satisfaction toward patient capacity and comprehension	No.	%
Were you educated about how to use the medication?	501	87.1%
Was the information provided to you about how to use the medication easy to understand?	517	89.9%
Were you able to ask your doctor to give you more information on the prescription?	389	67.7%
Were all your questions answered clearly?	483	84.0%
Mean ± SD	3.3 ± 1.0	
Overall percentage	82.5%	

A total of 517 (89.9%) patients agreed that the information provided to them about how to use the medication was easy to understand, 501 (87.1%) were educated about how to use the medication, and 483 (84%) reported that all their questions were answered clearly. The mean score was 3.3 ± 1.0 out of 4 (Table 3).

**Table 3:** The general satisfaction toward the physician and pharmacist among the study participants

The general satisfaction toward the physician and pharmacist	Disagree		Neutral		Agree	
	No.	%	No.	%	No.	%
Satisfied with the doctor's communication skills	39	6.8%	94	16.3%	442	76.9%
Satisfied with the doctor’s interest in improving your health condition or any complications and controlling the disease	67	11.7%	123	21.4%	385	67.0%
Satisfied with the necessary instructions and warnings given to you regarding your medicines (side effects, allergies), especially medicines that were prescribed for the first time	118	20.5%	115	20.0%	342	59.5%
Satisfied that the doctor adequately explained the duration of treatment (especially medicines that were prescribed for the first time)	68	11.8%	86	15.0%	421	73.2%
Satisfied with receiving medicines from the pharmacy exactly as it was prescribed	28	4.9%	54	9.4%	493	85.7%
Satisfied that the pharmacist has cooperated with the doctor in order to dispense the medicines to you correctly	34	5.9%	59	10.3%	482	83.8%
Satisfied that the pharmacist has tried to make sure you understand how to use your medication properly	69	12.0%	82	14.3%	424	73.7%
Satisfied that the pharmacist was polite and friendly	23	4.0%	52	9.0%	500	87.0%
Satisfied that the pharmacist explained how to take the medicines and why it is important to take them as directed	57	9.9%	87	15.1%	431	75.0%
Satisfied that the pharmacist always explains the side effects of medicines	212	36.9%	137	23.8%	226	39.3%
Satisfied that the pharmacist helped you when you had problems with your medicines	99	17.2%	148	25.7%	328	57.0%
Satisfied that the directions on your medication can be read easily	49	8.5%	70	12.2%	456	79.3%
Satisfied with the availability of the medicines or health devices you need	64	11.1%	82	14.3%	429	74.6%
Mean ± SD	50.7 ± 9.8					
Overall percentage	78%					

Moreover, 87% of the study patients were satisfied with the pharmacists, 85.7% were satisfied with receiving medicines from the pharmacy exactly as it was prescribed, 83.8% were satisfied that the pharmacist cooperated with the physicians to dispense the medicines to them correctly, 79.3% were satisfied that the directions on their medication can be read easily, 76.9% were satisfied with the doctor's communication skills, 75% were satisfied that the pharmacist explained how to take the medicines and why it is important to take them as directed, and 74.6% were satisfied with the availability of the medicines or health devices needed. Only 39.3% were satisfied that the pharmacist always explains the side effects of medicines. The overall mean score was 50.7 ± 9.8 out of 65 (Table 4).

**Table 4:** Distribution of self-perceived general satisfaction toward patient capacity by patient’s bio-demographic data

Bio-demographic data	Self-perceived general satisfaction toward patient capacity and comprehension		P-value
	Mean	SD	
Age in years			0.492
<25	3.38	0.94	
25-34	3.17	1.13	
35-44	3.27	1.00	
45-55	3.31	1.09	
>55	3.20	1.16	
Gender			0.032
Male	3.13	1.14	
Female	3.34	1.00	
Educational level			0.168
Below secondary	3.58	0.61	
Secondary	3.44	0.83	
University/above	3.25	1.08	
Number of received drugs			0.374
1-2	3.27	1.04	
3-5	3.43	0.99	
>5	3.11	1.32	
Duration of using these drugs			0.636
<7 days	3.26	1.08	
>7 days	3.31	1.01	

**Figure 1:**
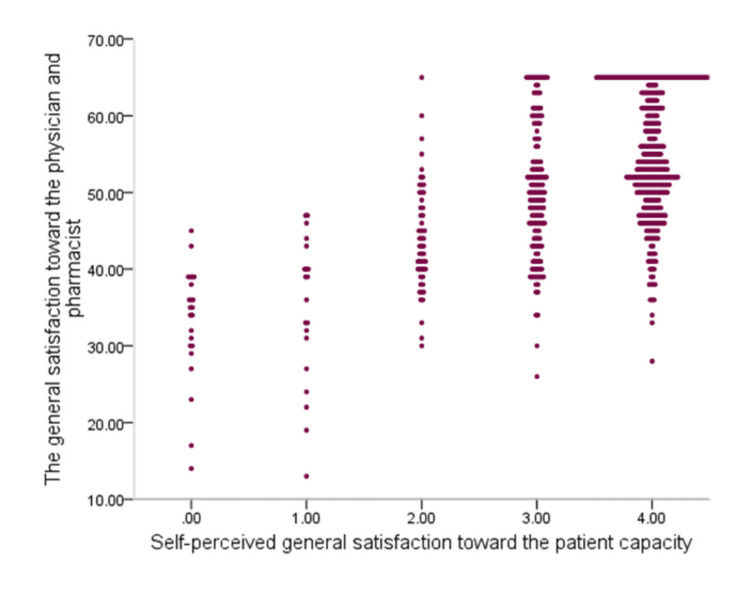
Scatter correlation of the general satisfaction toward the physician and pharmacist with self-perceived general satisfaction toward the patient capacity and comprehension

The mean satisfaction score was significantly higher among female patients (3.34 ± 1.0) than male patients (3.13 ± 1.14), with a statistically significant difference (p = 0.032). All other factors had no significant relation to patients’ satisfaction scores (Table 5). The mean satisfaction score was significantly higher among low-educated patients (54.7 ± 8.3) than among university-graduated patients (50.3 ± 10.0) with recorded statistical significance (p = 0.049). All other factors had no significant correlation with patients’ satisfaction scores. The graph (Figure 1) below shows a significant positive intermediate correlation between the two satisfaction domains (r = 0.57; p = 0.001).

**Table 5:** Distribution of the general satisfaction toward the physician and pharmacist by patient’s bio-demographic data

Bio-demographic data	The general satisfaction toward the physician and pharmacist and healthcare services		P-value
	Mean	SD	
Age in years			0.623
<25	51.4	10.4	
25-34	49.7	8.9	
35-44	50.9	9.2	
45-55	50.6	10.2	
>55	49.8	10.7	
Gender			0.725
Male	50.4	10.3	
Female	50.8	9.7	
Educational level			0.049
Below secondary	54.7	8.3	
Secondary	52.3	8.7	
University/above	50.3	10.0	
Number of received drugs			0.898
1-2	50.8	9.8	
3-5	50.3	10.1	
>5	50.0	10.5	
Duration of using these drugs			0.236
<7 days	51.2	10.2	
>7 days	50.2	9.5	

## Discussion

The purpose of this study was to find out how satisfied the participants were with their prescription and how well they understood it. Several studies have shown that patients often misinterpret explicit instructions because they do not even understand what their physicians are trying to say [11,12,23]. Our findings showed that 58 (10.1%) of participants of various ages had trouble comprehending the prescription provided to them by the physician. In a similar study, 38.6% of patients in four large hospitals in one city had a poor understanding of their prescriptions [11].

Participants were very satisfied with their ability to inquire and perceive information about their medications from their doctors' instructions, with the overall satisfaction of 3.3 ± 1.0 out of 4 and a maximum of 82.5%. However, only 32.3% were not able to inquire more about their prescription, which was the highest "No" answer in this domain; the reason for this could be that participants did not have an opportunity to do so.

Participants had a high level of understanding, with 87.1% indicating that the information for their prescription was very easy to understand. This might be due to effective physician communication and explanation of medication. Patients' characteristics are a crucial element in determining adherence. Elderly patients are more prone to have chronic conditions and use many medications, increasing medication complications. The majority of patients had more than one chronic disease, according to a recent cross-sectional study conducted in Saudi Arabia, and the pharmaceutical dose type and dosing schedule were independent predictors of medical adherence [22].

For effective patient care, a thorough investigation of this issue is necessary, and interventions such as specific content and format of prescription drug labels may assist patients in better understanding and remembering their medication [24]. Most participants were satisfied with their physicians' communication and the way doctors interact with patients, being informative and supportive, and how they show their interest in patients' health conditions, complications, or improvements. Effective communication is required for better quality healthcare systems, according to participants' attitudes toward physicians, pharmacists, and healthcare services. Participants were also satisfied with physicians' instructions, with physicians attempting to simplify information, especially for medications prescribed for the first time. However, the study found that 118 participants (20%) were unsatisfied with item warnings about medication, such as side effects and allergies, especially for medications received for the first time.

Patients must know what to anticipate from their treatments, and physicians should let them know if there are any risks. About 36.9% were unsatisfied with the way pharmacists routinely describe the adverse effects of any medication. Pharmacists, on the other hand, must guarantee that their patients not only get the right prescription and dosage but also that they are properly instructed on how to safely and successfully utilize their drug. The availability of drugs and health devices received a high satisfaction level of 74.6%. Female patients had a considerably higher mean satisfaction score than male patients with a statistically significant difference (p = 0.032). All other variables had no impact on the patients' satisfaction scores. Our findings revealed that gender varied considerably in prescription and self-perceived capacity to ask and communicate with the physician, with females being more satisfied (p = 0.032). On the other hand, another study showed that females are less likely to be satisfied with primary health care because they may have a higher level of expectation [20]. Furthermore, the educational level of the participants was shown to have a significant impact on patient satisfaction in this study. Lower educated participants were more satisfied (p = 0.049) with their self-perception of physicians, pharmacists, and health services, according to our findings. Patients who are well educated are more likely to have a better understanding of their diseases, treatment plans, and healthcare services in general. As a result, when their expectations were not met, they were unsatisfied. A recent study aimed at evaluating medication adherence and satisfaction among hypertensive patients demonstrated that the greater the education, the lower the satisfaction with healthcare services [17]. Several studies have also found a link between satisfaction and education levels [25,26].

Even though there was no significant correlation between the frequency of prescriptions and satisfaction in our study, it was found that patients often misunderstand regimen instructions [26]. Increased adherence may be achieved using medications that are designed to simplify or minimize dose. An electronic monitoring device was used to examine medication compliance in a systematic review study of 76 publications, and the authors found that fewer frequent dosage regimens resulted in higher compliance [27]. One potential explanation is that patients have a better comprehension of their pharmaceutical regimen when dosages per day are reduced, and regimen complexity is enhanced. However, since this subject is not well studied in the literature, we wanted to see how well participants comprehended their medicine. In our study, we have investigated how well participants understood their medications and how clear was their prescriptions, which has a high impact on patients’ perspectives and satisfaction with their medications.

The limitations of this study are that it should have included the weaker sections that are not versatile enough to use electronic form-filling facilities. Moreover, the sample was collected randomly through social media, without any specification to include location or region, which may induce sampling bias. However, we selected data collectors within different regions in Saudi Arabia and asked them to spread our questionnaire in their regions in an online-based manner. We also asked participants what they experienced retrospectively so we may introduce recall bias. We relied on self-reports from respondents speaking for themselves about drugs they had without associating them with their condition or type of medication or what type of faculty they visited.

## Conclusions

The current study demonstrated a high level of satisfaction among participants with healthcare services and both physicians and pharmacists in general. The majority of the participants were satisfied with the education they received, and they considered the information on how to take the drug to be simple to comprehend.

## Appendices

We are medical students from Umm Al-Qura University. We are doing research that aims to measure the patients' satisfaction and comprehension of physician and pharmacist prescriptions in Saudi Arabia.

Filling out the questionnaire will not take more than five minutes approximately.

We hope that you will participate in this research and answer the questions, knowing that all information will be confidential and will only be used for scientific research purposes and you can withdraw at any time before submitting the questionnaire.

1. Age: ☐ 14-24 years ☐ 25-35 years ☐ 36-45 years ☐46-55 years ☐ Above 55 years

2. Gender: ☐ Female ☐ Male

3. Educational level:

☐ College graduate or post-college

☐ Secondary

☐ Elementary + primary

4. Disease (for which participant visited primary health care)?

☐ Diabetes mellitus

☐ Hypertension

☐ Gastrointestinal diseases

☐ Cardiac and respiratory diseases

☐ Orthopedic problems

☐ Urinary tract infections

☐ Eye diseases

☐ Dermatological problems

☐ Oncological diseases

☐ Other diseases: ___

5. How many medications do you take?

☐ 1-2 ☐ 3-5 ☐ More than 5

6. For how long did you take the medications? 

☐ 7 days or less ☐ More than 7 days

7. Have you been educated about how to use the medication?

☐ Yes ☐ No

8. Was the information given to you on how to use the medication easy to understand?

☐ Yes ☐ No

9. Were you able to ask the doctor to clarify more about the prescription information?

☐ Yes ☐ No

10. Were all your questions answered clearly?

☐ Yes ☐ No

11. You were satisfied with the communication skills of the physician:

Strongly disagree

Disagree

Neutral

Agree

Strongly agree

12. You were satisfied by the interest of the physician in your medical conditions’ improvement or any complications and disease controlled?

Strongly disagree

Disagree

Neutral

Agree

Strongly agree

13. You were satisfied with the necessary instructions and warnings about your medications (side effects, allergy), especially for medications received for the first time?

Strongly disagree

Disagree

Neutral

Agree

Strongly agree

14. You were satisfied that the physician explains the treatment period sufficiently (especially when you receive medication for the first time)?

Strongly disagree

Disagree

Neutral

Agree

Strongly agree

15. You were satisfied that you receive the medications from the pharmacy exactly according to the prescription?

Strongly disagree

Disagree

Neutral

Agree

Strongly agree

16. You were satisfied that the pharmacist/staff consult and cooperate with the physician for the correct dispensing of medicines to you?

Strongly disagree

Disagree

Neutral

Agree

Strongly agree

17. You are satisfied that the pharmacist or staff tries to make sure that you understand how to take your medications properly?

Strongly disagree

Disagree

Neutral

Agree

Strongly agree

18. You were satisfied that the pharmacist was polite and friendly?

Strongly disagree

Disagree

Neutral

Agree

Strongly agree

19. You were satisfied that the pharmacist explains how to take the medications and why it is important to take medications as directed?

Strongly disagree

Disagree

Neutral

Agree

Strongly agree

20. You were satisfied that the pharmacist always explains the side effects of medications?

Strongly disagree

Disagree

Neutral

Agree

Strongly agree

21. You are satisfied that the pharmacist is helpful when you have problems with medications?

Strongly disagree

Disagree

Neutral

Agree

Strongly agree

22. You are satisfied that the instructions on your medications are easily readable?

Strongly disagree

Disagree

Neutral

Agree

Strongly agree

23. You are satisfied with the availability of medicines or health appliances you need?

Strongly disagree

Disagree

Neutral

Agree

Strongly agree
